# Distinct neural responses of ventromedial prefrontal cortex-projecting nucleus reuniens neurons during aversive memory extinction

**DOI:** 10.1186/s13041-025-01185-y

**Published:** 2025-03-05

**Authors:** Yuki Mochizuki, Asuka Joji-Nishino, Kazuo Emoto, Akira Uematsu

**Affiliations:** 1 Department of Biological Sciences, Graduate School of Science, The University of Tokyo, Tokyo, Japan; 2 Human Informatics and Interaction Research Institute, National Institute for Advanced Industrial Science and Technology, Tsukuba, Japan; 3 Department of Animal Resource Sciences, Graduate School of Agricultural and Life Sciences, The University of Tokyo, Tokyo, Japan; 4 International Research Center for Neurointelligence (WPI-IRCN), The University of Tokyo, Tokyo, Japan

**Keywords:** Mediodorsal thalamic nucleus, Reuniens thalamic nucleus, Extinction, Aversive memory

## Abstract

Animals adaptively regulate aversive memories in safe environments through extinction, a process central to exposure therapy for anxiety disorders. The limbic thalamus controls cognitive function in concert with interconnected cortical and limbic structures. Though medial prefrontal (mPFC) afferents to the limbic thalamus regulate aversive memory, the functional role of limbic thalamus efferents to mPFC is unclear. Here, we investigated the roles of thalamic nuclei, the reuniens (RE) and mediodorsal (MD) thalamus, projecting to the medial prefrontal cortex (mPFC) in aversive memory conditioning and extinction in male mice. Using retrograde tracing, we demonstrated that ventromedial PFC (vmPFC)- and dorsomedial PFC (dmPFC)-projecting neurons are topologically segregated within the RE and MD. Fiber photometry revealed that both RE→vmPFC and MD→vmPFC neurons respond to aversive stimuli. Notably, RE→vmPFC neurons develop shock-associated cue (CS+) response during aversive conditioning. During extinction, RE→vmPFC neurons exhibited a biphasic response to CS+, while MD→vmPFC neurons showed no cue-evoked activity. Neither optogenetic activation nor inactivation of these populations altered freezing behavior during extinction compared to controls. Collectively, these findings indicate that RE→vmPFC neurons encode aversive cue information during extinction but are dispensable for behavioral modulation. This study highlights the distinct contributions of limbic thalamus-PFC circuits to aversive memory processing.

**Supplementary Information:**

The online version contains supplementary material available at 10.1186/s13041-025-01185-y.

## Introduction

Animals need to predict threats and respond appropriately to cues and situations that indicate danger for their survival. However, when the cue is repeatedly presented in a safe environment, they can adaptively suppress aversive memory as threat-predicted cues are no longer associated with danger [1]. This adaptive process, called extinction, is the basis of exposure therapy to treat anxiety disorders by gradually diminishing maladaptive fear responses [2]. Thus, understanding the neural basis of these processes can advance therapeutic strategies for anxiety disorders [3, 4].

In the laboratory, cued aversive conditioning is commonly studied in rodents by pairing a conditioned stimulus (CS) with an aversive unconditioning stimulus (US). For aversive memory extinction, CS is repeatedly presented without US, resulting in a gradual reduction of conditioned defensive responses [5]. The neural circuits underlying aversive conditioning and extinction primarily involve the medial prefrontal cortex, amygdala, and ventral hippocampus, which are directly or indirectly interconnected and function in concert to mediate these processes [6–15]. Each brain region is subdivided into several areas which have specific functions in regulating aversive conditioning and extinction. For example, within the mPFC, the dorsomedial prefrontal cortex (dmPFC) is important for expression of defensive behavior, whereas the ventromedial prefrontal cortex (vmPFC) is crucial for extinction [16–21].

The thalamic nuclei are anatomically connected with mPFC, ventral hippocampus, and amygdala, serving as relays to mediate indirect connections among these brain regions. Accumulating evidence indicates that the limbic thalamus plays a crucial role in cognitive functions by acting as a hub that modulates information flow between cortical and subcortical regions and/or enhancing functional cortical connectivity [22, 23]. In particular, the nucleus reuniens (RE) and mediodorsal thalamic (MD) nuclei play essential roles for aversive memory extinction [24–29]. Pharmacological inactivation of the RE impairs aversive extinction or retrieval. Similarly, knockdown of phospholipase C beta4 in the MD results in extinction deficits accompanied by enhanced burst firing in MD neurons. Circuit-wise, RE neurons projecting to the ventral hippocampus encodes memory of context- and cue-dependent extinction learning [30]. The RE input to basolateral amygdala engages in extinction of remote aversive memory [31]. Additionally, the vmPFC afferents to the RE regulate conditioned defensive responses [26, 32]. Despite the dense reciprocal connections of both RE and MD with the mPFC, the functional roles of RE or MD neurons projecting to vmPFC in aversive memory conditioning and extinction remains largely unexplored. The present study aimed to shed light on this question using a combination of retrograde tracing, fiber photometry, and optogenetics.

## Methods

### Animals

All procedures were conducted in accordance with the guidelines set by the Animal Care Committee of the University of Tokyo and the National Institute of Advanced Industrial Science and Technology (AIST). Male C57BL/6 J mice, aged 8–12 weeks at the time of surgery, were obtained from Japan SLC, Inc. or CLEA Japan, Inc. All behavioral experiments were conducted during the light phase.

### Surgery

All surgeries were performed under aseptic conditions using isoflurane anesthesia (Viatris, 3% for induction, 1% for maintenance at 0.3 L/min), as previously described [33]. Mice were positioned using a stereotaxic apparatus.

For retrograde tracing, 300 nL of Cholera Toxin Subunit B (CTB)(Recombinant), Alexa Fluor™ 488 Conjugate (Invitrogen™) and Cholera Toxin Subunit B (Recombinant), Alexa Fluor™ 555 Conjugate (Invitrogen™) were injected unilaterally into the dorsomedial prefrontal cortex (dmPFC) (AP: +2.00 mm, ML: ±0.30 mm, DV: +2.00 mm) and ipsilateral ventromedial prefrontal cortex (vmPFC) (AP: +1.80 mm, ML: ±1.72 mm, DV: +2.47 mm, angle 30°). CTB Alexa Fluor™ 488 Conjugate and CTB Alexa Fluor™ 555 Conjugate injections were counterbalanced among the subjects. Mice were allowed to recover in their home cages for 2 weeks post-surgery.

For fiber photometry experiments, 300 nL of retroAAV-hSyn-Cre (Addgene, 105553, titer: 2.1 × 10e13 vg/ml) was injected into the vmPFC using the coordinates mentioned above. Additionally, AAV1-hSyn-Flex-jGCaMP7b (Addgene, 104493, titer: 1.9 × 10e13 vg/ml) was injected into the ipsilateral RE (AP: -1.35 mm, ML: ±2.60 mm, DV: -3.70 mm, angle 30°) and the MD (AP: -1.70 mm, ML: ±0.30 mm, DV: -3.30 mm). Optic fibers were implanted in the RE and MD using the same coordinates, with the MD implantation site at AP: -1.70 mm, ML: ±1.04 mm, DV: -2.97 mm, angle 10°. Optic fibers were secured using UV glue (Bondic, BD-CRJ), Super-Bond (San Medical), and acrylic dental cement (YAMAHACHI DENTAL MFG., CO., Re-fine Bright).

For optogenetic inhibition experiments, 300 nL of retroAAV-hSyn-Cre (Addgene, 105553, titer: 2.1 × 10e13 vg/ml) was injected bilaterally into the vmPFC, and 300 nL of AAV5-hSyn-Flex-Jaws-KGC-GFP-ER2 (UNC Vector Core, 6.1 × 10e12 vg/ml) or AAV5-hSyn-DIO-EGFP (Addgene, 50457, 6.5 × 10e12 vg/ml) was injected bilaterally into the RE (AP: +1.50 mm, ML: ±1.10 mm, DV: -4.50 mm, angle 10°) or MD (AP: -1.70 mm, ML: ±0.30 mm, DV: -3.30 mm). Optic fibers were implanted bilaterally above the RE (AP: -1.50 mm, ML: ±1.20 mm, DV: -3.90 mm, angle 10°) or MD (AP: -1.70 mm, ML: ±1.20 mm, DV: -2.55 mm, angle 10°).

For optogenetic activation experiments, 300 nL of retroAAV-hSyn-Cre (Addgene, 105553, titer: 2.1 × 10e13 vg/ml) was injected into the bilateral vmPFC, and 300 nL of AAV5-hSyn-Flex-ChrimsonR-tdTomato (Addgene, 59171, 1.0 × 10e12 vg/ml) or AAV5-hSyn-DIO-mCherry (Addgene, 50459, 5.3 × 10e12 vg/ml) was injected into the bilateral RE or MD. Optic fibers were then implanted bilaterally above the RE or MD using the same coordinates as those used for the optogenetic inhibition experiments.

### Fiber photometry

All behavioral experiments were conducted in a sound-isolated box located within a soundproof room. The auditory conditioned stimulus (CS) consisted of two distinct tones: CS+ and CS− (4 or 10kHz, 74 dB, 20 s, counterbalanced across subjects), and the unconditioned stimulus (US) was a 0.4 mA footshock (2s). The CS+ was paired with the US, whereas the CS- was presented without the US, serving as a control stimulus to assess associative learning. The US was initiated immediately after the CS ended. For fiber photometry experiments, the timings of CS and US were presented using a conditioning apparatus (O’Hara & Co., Ltd.) controlled by Bonsai (https://bonsai-rx.org).

Fiber photometry recordings were conducted using the FP3002 system (Neurophotometrics), controlled by Bonsai. The 470 nm and 415 nm (isosbestic) LEDs in the FP3002 system were calibrated to deliver 100 µW of power at the tip of the fiber, with recordings taken at a 10 Hz sampling rate for each wavelength. On Day 0, mice were habituated by presenting them with 30 tones (4–10 kHz, 20 s, CS-) in their home cage, with an inter-trial interval (ITI) averaging 30 s. On Day 1, mice underwent aversive memory conditioning, receiving 5 CS- and 5 pairings of CS+ and US with a random ITI average 90 s. On Day 2, mice were presented with 5 CS- and 30 CS+ in the extinction context (ITI: average 70 s). On Day 3, mice received 5 CS- and 10 CS+ in the extinction context (ITI: average 70 s). Freezing rates were automatically analyzed using DeepLabCut (https://github.com/DeepLabCut/DeepLabCut) and MATLAB.

### Aversive memory extinction with optogenetic manipulations

The red laser was generated using a diode-pumped solid 635 nm laser solid-laser. The laser was calibrated to deliver over 5 mW (for MD inhibition) or 10 mW (for other conditions) at the tip of the fiber. On Day 1, mice received 5 CS (4 kHz) presentations immediately followed by US. On Day 2, mice were presented with 40 CS (for MD inhibition) or 30 CS (for other conditions). On Day 3, mice received 6 CS (ITI: average 70 s). For inhibition studies, laser illumination was initiated 400 ms before the CS onset and lasted until 3 s of the CS offset during extinction. For activation studies, 10 Hz laser stimulation was delivered during the CS period of extinction. Freezing rates were automatically analyzed using DeepLabCut (https://github.com/DeepLabCut/DeepLabCut) and a custom-code on MATLAB.

### Histology

Following the behavioral experiments, mice were anesthetized with isoflurane and perfused with 10 mL of 0.1 M PBS followed by 30 mL of 4% paraformaldehyde in 0.1 M PBS. The brains were extracted, post-fixed, and then submerged in 30% sucrose PBS (for retrograde tracing and optogenetic activation). The brains were sectioned into 40 μm coronal slices using a cryostat (for retrograde tracing and optogenetic activation) or a VT1200S (Leica) (for optogenetic inhibition). For CTB retrograde experiments, images of labeled cells were acquired using a dragonfly confocal microscopy system (Oxford Instruments). The regions of RE and MD were defined according to Paxinos and Watson Brain Atlas. The number of retrogradely labeled neurons and their locations in these areas were quantified with custom-written cell count programs using Fiji (https://imagej.net/software/fiji/). Image acquisition and counts were performed in every third section. For fiber photometry and optogenetic experiments, the placement of optic fibers and the expression of the virus were confirmed by examining the slices under a BZ-X800 microscope (KEYENCE).

### Data and statistical analysis

Fiber photometry data were processed for normalization, fitting, and sorting using a custom written program in MATLAB 2021 (https://jp.mathworks.com/products/matlab.html). Motion correction was performed by adjusting the fluorescence of GCaMP7b measured at 470 nm with data from the 410 nm isosbestic point. The Z-score was calculated by first determining the mean and standard deviation of the 20 s preceding the CS. Value at each time point was then normalized by subtracting the mean and dividing by the standard deviation.

All statistical analyses were conducted using GraphPad Prism (GraphPad Software, Inc.) and Matlab. The data were subjected to the Shapiro-Wilk normality test, and the appropriate statistical methods were selected and applied based on the results. After sphericity test, ANOVA tests were adjusted a priori with Geisser-Greenhouse correction in order to avoid violating sphericity assumption [34].

## Results

### Neurons projecting to dmPFC or vmPFC are distinct in the RE and MD

**Fig. 1:**
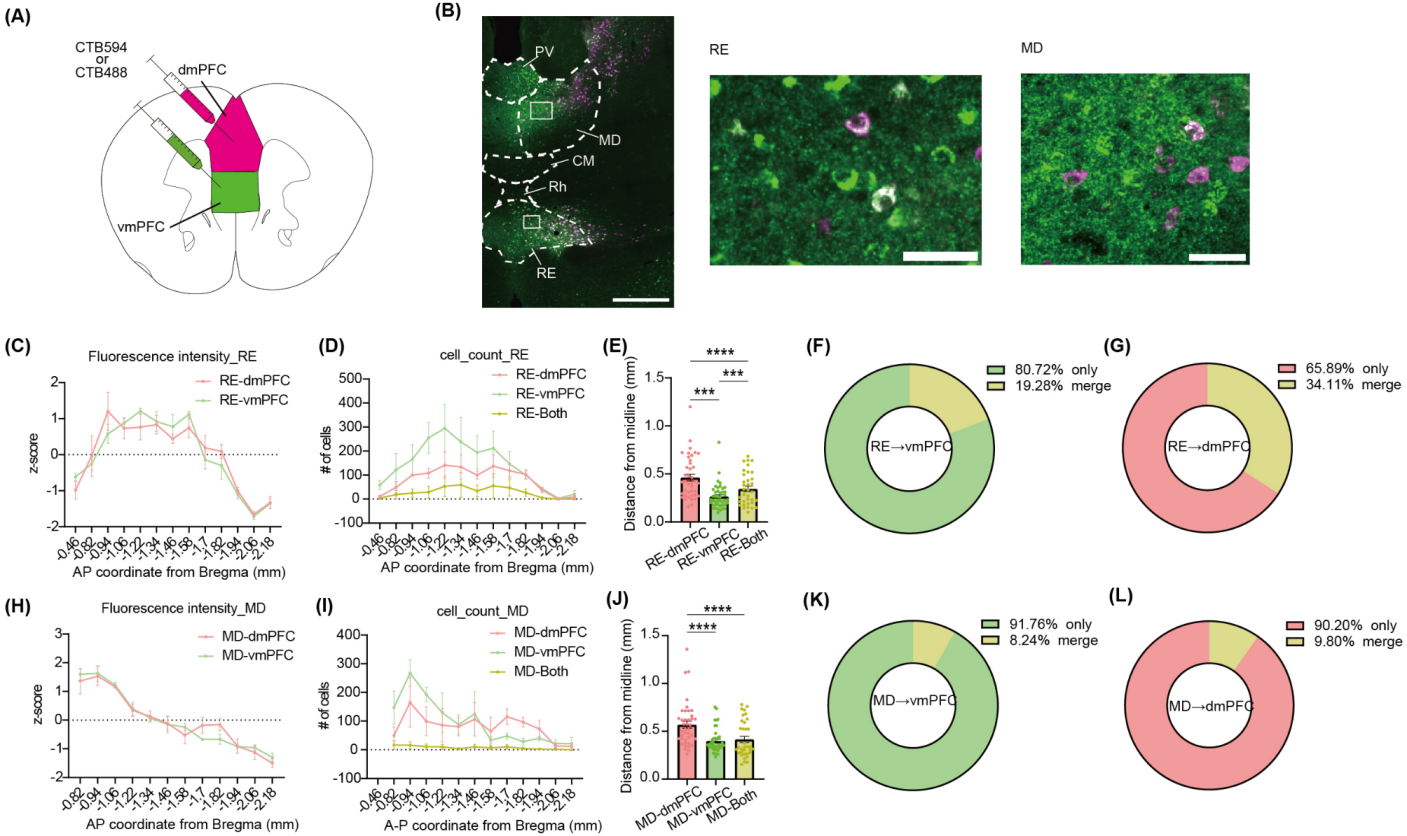
Distinct neural populations which project to dmPFC or vmPFC in the limbic thalamus. **(A)** Schematic image of the retrograde tracing surgery. **(B)** Representative images of CTB tracing (Left: whole midline thalamus, white bar = 500 μm, Middle: magnified image in RE, white bar = 50 μm, right: magnified image in MD). **(C**, **H)** Mean fluorescent intensity in **(C)** RE or **(D)** MD along the AP axis (*n* = 3 mice). **(D**, **I)** The mean number of labeled cells in **(D)** RE or **(I)** MD along the AP axis. **(E**, **J)** The mean position of individual labeled cells from the midline including all AP slides in the **(E)** RE (Mixed-effects analysis, *F*(1.91, 68.8) = 52.48, *p* < 0.0001, post-hoc Sidak’s multiple comparisons test RE-dmPFC vs. RE-vmPFC *p* < 0.0001, RE-dmPFC vs. RE-Both *p* < 0.0001, RE-vmPFC vs. RE-Both *p* = 0.0001), or **(J)** MD (Mixed-effects analysis, *F*(1.43, 50.63) = 20.42, *p* < 0.0001, post-hoc Sidak’s multiple comparisons test MD-dmPFC vs. MD-vmPFC *p* < 0.0001, MD-dmPFC vs. MD-Both *p* < 0.0001, MD-vmPFC vs. MD-Both *p* = 0.81). **(F**, **G)** The ratio of RE neurons projecting to both vmPFC and dmPFC relative to those projecting to **(F)** vmPFC or **(G)** dmPFC. **(K**, **L)** The ratio of MD neurons projecting to both vmPFC and dmPFC relative to those projecting to **(K)** vmPFC or **(L)** dmPFC. All error bars indicate SEM. ***p < 0.001, ***p < 0.0001

To identify thalamic neurons projecting to the dmPFC or vmPFC, we injected distinctly colored CTBs into the dmPFC and vmPFC (Fig. 1A). Neurons projecting to the dmPFC were predominantly found in the lateral part of the MD. In contrast, neurons projecting to the vmPFC were located not only in the ventral RE but also in the medial division of the MD (Fig. 1B). To further validate the topological independence of these populations, we analyzed the spatial distribution of individual neurons. In the RE, both dmPFC-projecting neurons and vmPFC-projecting neurons were localized in the middle part along the A-P axis (Fig. 1C and D). Notably, neurons projecting to the vmPFC were concentrated in the medial region of the RE compared to dmPFC-projecting neurons. These neural populations exhibited minimal overlap (Fig. 1E-G). In the MD, both dmPFC-projecting neurons and vmPFC-projecting neurons were mainly located at the anterior part (Fig. 1H and I). Similar to the RE, neurons projecting to the vmPFC are located more medially in the MD than those projecting to the dmPFC (Fig. 1E). In addition, a small subset of neurons projected to both the dmPFC and vmPFC (Fig. 1J-L). Together, these findings demonstrate that dmPFC- and vmPFC-projecting populations in the limbic thalamus are topologically segregated.

### RE→vmPFC, but not MD→vmPFC neurons, show increased response to CS+ during aversive conditioning

**Fig. 2:**
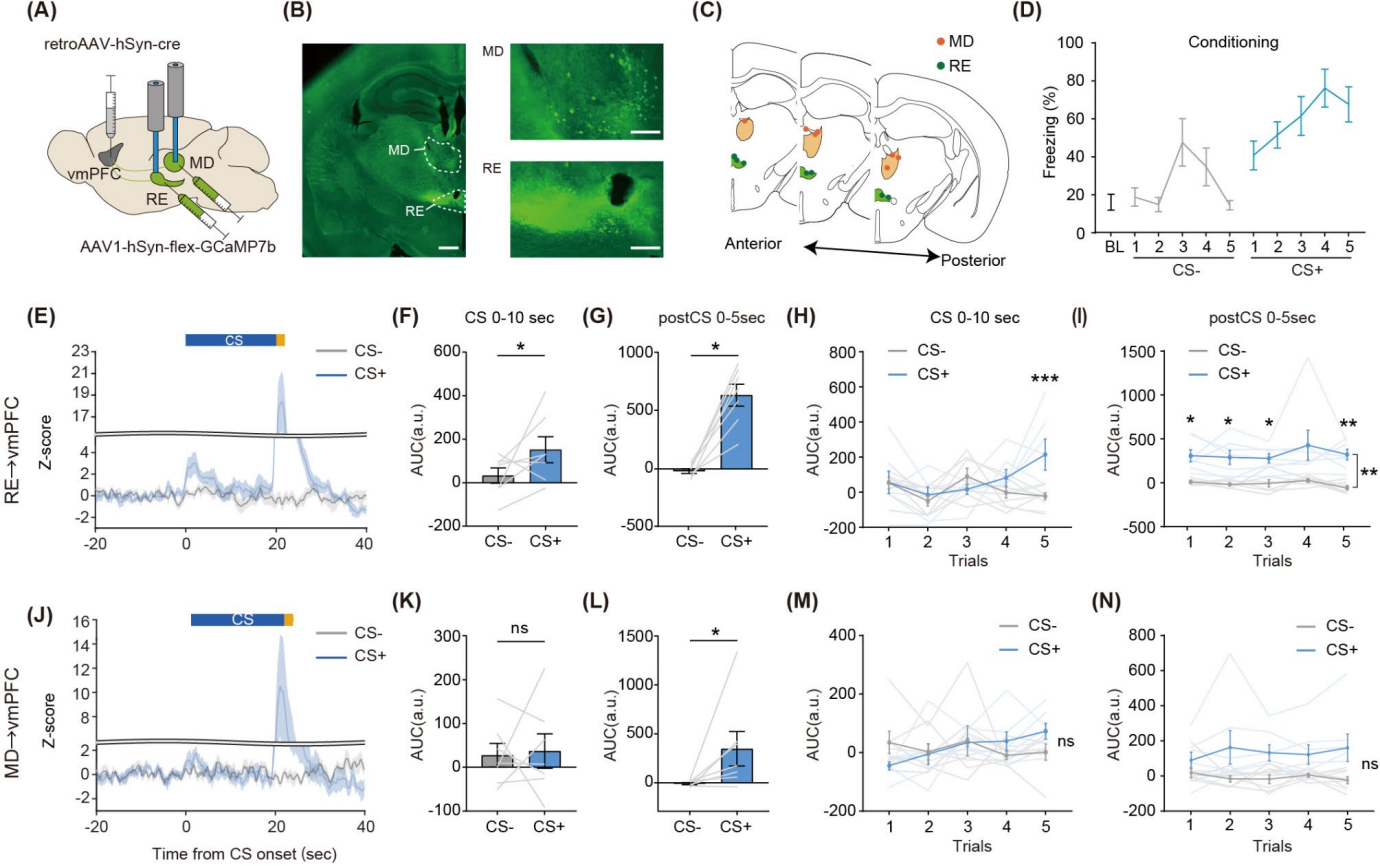
Shock evoked response of vmPFC-projecting RE and MD neurons. **(A)** Schematic of multi-fiber photometry recording from the RE and MD. The fibers were implanted above the RE and MD. **(B)** Representative image showing GCaMP expression (Left: whole midline thalamus, white bar = 500 μm, Right: magnified images, white bar = 200 μm) **(C)** Green and orange circle indicates sites of implanted optic fibers above the RE and MD, respectively. **(D)** Mean percentage of freezing during each tone throughout aversive conditioning (*n* = 7 mice). **(E**, **J)** Mean Z-scored calcium activity of vmPFC-projecting **(E)** RE or **(J)** MD neurons from a population of mice in response to CS+ or CS- during aversive conditioning. Shaded region denotes standard mean errors. At the top of each plot, blue and orange lines denote CS and US period, respectively (*n* = 7 for each). **(F**, **K)** Mean Z-score AUC during the 10 s after CS onset in the **(F)** RE (paired t-test, *t* (6) = 2.65, *p* = 0.027) or **(K)** MD (paired t-test, *t* [6] = 1.33, *p* = 0.22). **(G**, **L)** Mean Z-score AUC during the 5 s after CS offset in **(G)** RE (Wilcoxon’s signed rank test, *W* = 28.0, *p* = 0.016) or **(L)** MD (Wilcoxon’s signed rank test, *W* = 26.0, *p* = 0.031). **(H**, **M)** Mean Z-score of the AUC for each trial during the 10 s after CS onset in **(H)** RE (interaction: *F* (4, 24) = 4.01, *p* = 0.012, main effect of CS type: *F* (1, 6) = 3.34, *p* = 0.12) and **(M)** MD (interaction: *F*(1.92,11.53) = 1.64, *p* = 0.33, main effect of CS type: *F* (1, 6) = 0.07, *p* = 0.80). **(I**, **N)** Mean Z-score of the AUC for each trial during the 5 s after CS offset in the **(I)** RE (interaction: *F*(1.72,10.32) = 0.43 *p* = 0.63; main effect of CS type: *F* (1, 6) = 26.53, *p* = 0.0021) and **(N)** MD (interaction: *F*(1.85,11.09) = 1.29, *p* = 0.31, main effect of CS type: *F* (1, 6) = 5.14, *p* = 0.064). Shaded lines depict data from individual subjects. All error bars indicate SEM across subjects. (**H**, **I**,**M**, **N**) two-way repeated measures ANOVA followed by Sidak’s multiple comparison test. *p < 0.05, ***p < 0.001

Next, we sought to observe neural response of vmPFC-projecting neurons in RE or MD during aversive memory formation. To achieve this, we injected retroAAV-Cre into the vmPFC followed by injections of AAV carrying a construct encoding a Cre-dependent GCaMP7b protein into both RE and MD (Fig. 2A). We then simultaneously monitored bulk Ca^2^^+^ signals from RE and MD neurons projecting to the vmPFC using multi-fiber photometry (Fig. 2B and C). After a habituation session, mice were subjected to a differential aversive conditioning paradigm (Fig. 2D, day 1) in which one of two tones (CS+) was paired to a footshock (unconditioned stimulus; US) whereas a second tone (CS−) was not. Mice showed the significantly higher levels of freezing to the CS+ compared to the CS- (two-way repeated measures ANOVA, *trial×CS type*: *F*(2.21,13.25) = 1.40, *p* = 0.28; main effect of CSs: *F* (1, 6) = 136.6, *p* < 0.0001).

Multi-fiber photometry revealed that both vmPFC-projecting RE and MD neurons were activated by foot shock (Fig. 2G and L, Wilcoxon’s matched-pairs signed rank test, RE: *W* = 28.0, *p* = 0.016; MD: *W* = 26.0, *p* = 0.031). In the average of the entire conditioning session, CS+ evoked response is prominent at the first 10 s after CS onset, we assessed the area under the curve (AUC) of bulk Ca^2 +^ response during this period (Fig. 2E and J). We found that AUC for the CS+ was significantly higher compared to the CS- in the RE (Fig. 2F, *t* (6) = 2.65, *p* = 0.027), but not in the MD (Fig. 2K, t (6) = 1.33, *p* = 0.22). Next, we tested whether CS+ activity in the RE-vmPFC might develop during learning. A two-way repeated measures ANOVA found a significant effect of the interaction between CSs and trial number (Fig. 2H, F (4, 24) = 4.01, *p* = 0.012). Post-hoc Sidak’s multiple comparison test revealed a significant increase in neural activity during the CS+ compared to the CS- on the fifth trial (*p* = 0.0020), suggesting CS+ evoked responses in RE-vmPFC neurons developed in later trials by aversive learning. Notably, vmPFC-projecting RE neurons did not exhibit CS-evoked responses during the habituation phase (Supplementary Fig. 1), supporting that the CS+ responses observed during the conditioning phase are a result of associative learning. These results indicate that both RE and MD neurons convey aversive US signals to the vmPFC and that vmPFC-projecting RE neurons send aversive cue information.

### RE→vmPFC neurons exhibited a biphasic response to CS+ during the early phase of extinction

**Fig. 3:**
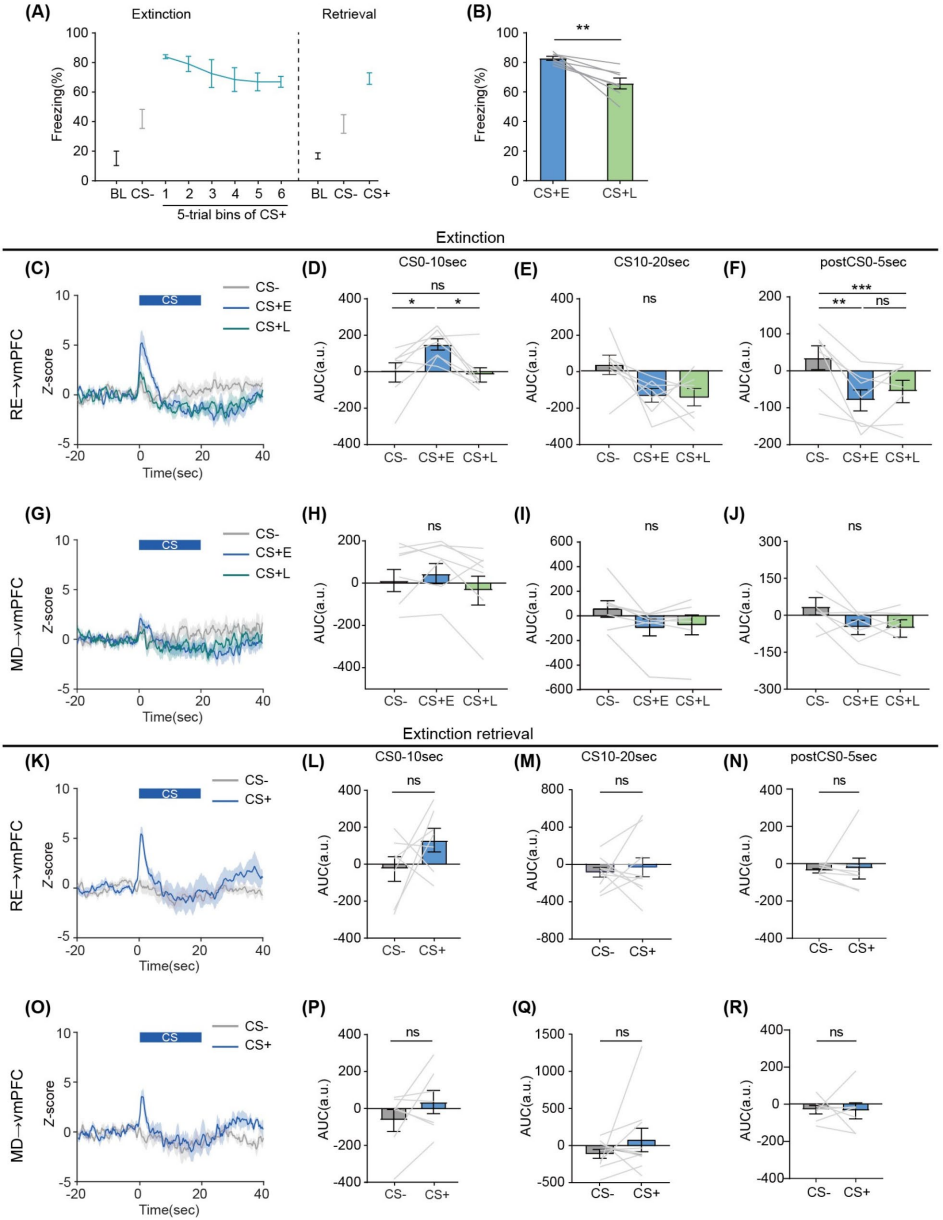
Biphasic response of vmPFC-projecting RE neurons in response to aversive cue at the early phase of aversive extinction. **(A)** Mean percentage of freezing throughout aversive extinction and retrieval. BL denotes the baseline period, which is 20 s before the first CS- trial. Each point represents the averaged block of 5 trials. **(B)** Mean freezing during the early phase (CS+ E) and the late phase (CS+ L) of extinction (paired t-test, *t* [6] = 4.23, *p* < 0.01). **(C**, **G)** Mean Z-scored calcium activity of vmPFC-projecting **(C)** RE or **(G)** MD neurons from a population of mice in response to CSs during aversive extinction. Shaded line indicates the standard error of the mean. At the top of each plot, the blue line denotes the CS period. **(D**, **H)** Mean Z-score AUC during the 10 s after CS onset in **(D)** RE (*F*(1.92,11.54) = 7.79, *p* < 0.01) or (H) MD (*F*(1.85,11.11) = 2.19, *p* = 0.16). **(E**, **I)** Mean Z-score during the last 10 s of CS in **(E)** RE (*F*(1.70,10.32) = 4.02, *p* = 0.056) or **(I)** MD (*F*(1.17,7.00) = 4.25, *p* = 0.075). **(F**, **J)** Mean Z-score during the 5 s after CS offset in **(F)** RE (*F*(1.19, 7.13) = 18.51, *p* < 0.001) or **(J)** MD (*F*(1.55, 9.31) = 4.66, *p* = 0.046). **(K**, **O)** Mean Z-scored calcium activity of vmPFC-projecting **(K)** RE or **(O)** MD neurons from a population of mice in response to CS+ or CS- during retrieval. **(L**, **P)** Mean Z-score AUC during the 10 s after CS onset in the **(L)** RE (paired t-test, *t* (6) = 1.34, *p* = 0.23) or **(P)** MD (Wilcoxon’s signed rank test, *W* = 12.0, *p* = 0.38). **(M**, **Q)** Mean Z-score during the last 10 s of CS in the (M) RE (Wilcoxon’s signed rank test, *W* = -4.0, *p* = 0.22) or **(Q)** MD (Wilcoxon’s signed rank test, *W* = 6.0, *p* = 0.69). **(N**, **R)** Mean Z-score during the 5 s after CS offset in **(N)** RE (Wilcoxon’s signed rank test, *W* = -14.0, *p* = 0.30) or **(R)** MD (paired t-test, *t* (6) = 0.11, *p* = 0.92). Shaded lines depict data from individual subjects. All error bars indicate SEM across subjects (*n* = 7 mice). **(D)**-**(J)** one-way repeated measures ANOVA followed by Tukey’s multiple comparison test. *p < 0.05, **p < 0.01, ***p < 0.001

Animals were then subjected to extinction learning and retrieval, where the CS+ were repeatedly presented without shock. Freezing was significantly higher for the CS+ at the early extinction trials (CS+ E; first 5 trials) than for the CS- (Fig. 3A, Dunn’s multiple comparisons test, CS- vs. CS+ 1–5 trials: *p* < 0.001, CS- vs. CS+ 6–10 trials: *p* < 0.001, CS- vs. CS+ 11–15 trials: *p* < 0.01, CS- vs. CS+ 16–20 trials: *p* = 0.38, CS- vs. CS+ 21–25 trials: *p* = 0.21, CS- vs. CS+ 26–30 trials: *p* = 0.38). Also, the freezing during the CS+ at the late extinction trials (CS+ L; last 5 trials) was significantly lower than that during the CS+ E (Fig. 3B, paired t-test, *t* (6) = 4.23, *p* < 0.01), indicating that mice can successfully extinguish the aversive memory.

During extinction, vmPFC-projecting neurons in the RE or MD exhibited a biphasic response to CS+, characterized by increased Ca^2+^ responses after CS+ onset subsequently followed by a suppressed response (Fig. 3C and G). To quantify these dynamics, we calculated the AUC of Ca^2+^ activity during the first 10 s of CS presentation, the subsequent 10 s of CS presentation, and the 5 s following CS offset at each CS phase. During the first 10 s of CS presentation, one-way repeated measures ANOVA found a significant main effect of CS type in vmPFC-projecting RE neurons (*F*(1.92,11.54) = 7.79, *p* < 0.01). Tukey’s post-hoc test indicated a significantly higher AUC at the CS+ E and CS+ L compared to the CS- (Fig. 3D, CS- vs. CS+ E: *p* < 0.05, CS- vs. CS+ L: *p* = 0.96, CS+ E vs. CS+ L: *p* < 0.05). During the following 10 s of CS presentation, one-way repeated measures ANOVA found no significant difference in CS type of vmPFC-projecting RE neurons (*F*(1.70,10.32) = 4.02, *p* = 0.056). For the suppressed response after CS offset, one-way repeated measures ANOVA revealed a significant main effect of CS type (*F*(1.19, 7.13) = 18.51, *p* < 0.001). Tukey’s post-hoc test showed a significant decrease in AUC for both the CS+ E and CS+ L compared to the CS- (Fig. 3F, CS- vs. CS+ E: *p* < 0.01, CS- vs. CS+ L: *p* < 0.001, CS+ E vs. CS+ L: *p* = 0.53). In contrast, we failed to detect any significant difference in AUC after CS onset (Fig. 3H, *F*(1.85,11.11) = 2.19, *p* = 0.16) or during the subsequent CS period (Fig. 3I, *F*(1.17,7.00) = 4.25, *p* = 0.075) in vmPFC-projecting MD neurons. For the AUC during the 5 s from CS offset, one-way repeated measures ANOVA found a significant main effect of CS type (*F*(1.55, 9.31) = 4.66, *p* = 0.046) but post-hoc test couldn’t find any significant differences (Fig. 3J, CS- vs. CS+ E: *p* = 0.12, CS- vs. CS+ L: *p* = 0.13, CS+ E vs. CS+ L: *p* = 0.98). Additionally, we observed AUC during the first 10 s of CS presentation gradually decreases over extinction in vmPFC-projecting RE neurons, though we couldn’t detect any statistical differences (supplementary Fig. 2).

During extinction retrieval, vmPFC-projecting neurons in the RE or MD again exhibited a biphasic response to the CS+ (Fig. 3K and O). However, there were no significant differences between AUCs of the CS+ and CS- during the first 10 s of CS, the last half of CS, or the 5 s after CS offset in vmPFC-projecting RE neurons (Fig. 3L, paired t-test, *t* (6) = 1.34, *p* = 0.23; Fig. 3M, Wilcoxon’s matched-pairs signed rank test, *W* = -4.0, *p* = 0.22; Fig. 3N, Wilcoxon’s matched-pairs signed rank test, *W* = -14.0, *p* = 0.30). Likewise, in vmPFC-projecting MD neurons, no significant differences were observed between AUCs of the CS+ and CS- during all CS phases (Fig. 3P, Wilcoxon’s matched-pairs signed rank test, *W* = 12.0, *p* = 0.38; Fig. 3Q, Wilcoxon’s matched-pairs signed rank test, *W* = 6.0, *p* = 0.69; Fig. 3R, paired t-test, *t* [6] = 0.11, *p* = 0.92). Together, these results indicate that vmPFC-projecting RE neurons show a biphasic response to CS+ during the early phase of extinction.

### No significant changes in freezing behavior were observed by optogenetic manipulation of vmPFC-projecting RE or MD neurons

**Fig. 4:**
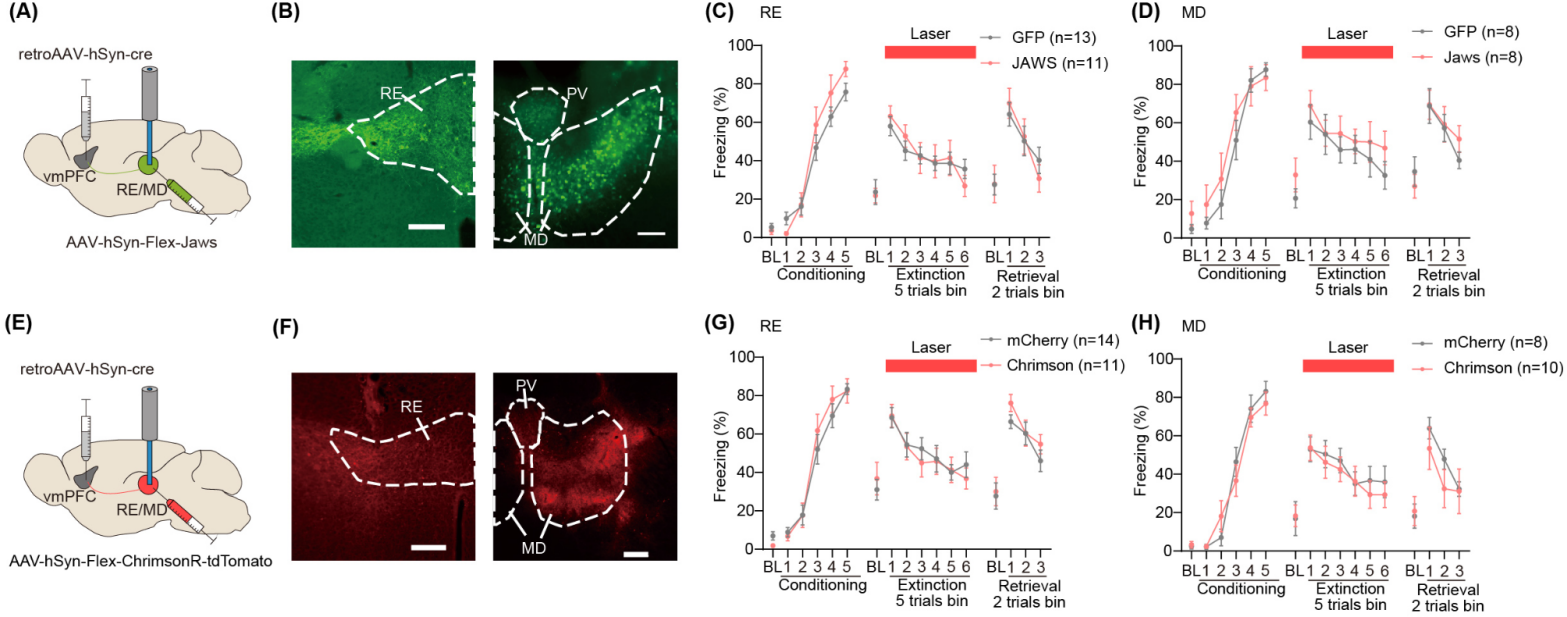
Optogenetic manipulation of vmPFC-projecting neurons in the RE or MD. **(A)** Schematic of retroAAV-Cre injection into the vmPFC, combined with AAV-Flex-Jaws-GFP or AAV-DIO-GFP injection into the RE or MD. **(B)** Representative images showing Jaws-GFP expression. White bar = 200 μm. **(C**, **D)** Inhibition of vmPFC-projecting **(C)** RE (Extinction, interaction, *F*(5, 95) = 1.29, *p* = 0.28; main effect of group, *F* (1, 19) = 0.024, *p* = 0.88. Extinction retrieval, interaction, *F* (2, 38) = 2.25, *p* = 0.12; main effect of group, *F* (1, 19) = 0.0023, *p* = 0.96) or **(D)** MD (Extinction, interaction, *F*(5, 70) = 0.84, *p* = 0.52; main effect of group, *F* (1, 14) = 0.47, *p* = 0.50. Extinction retrieval, interaction, *F* (2, 28) = 0.072, *p* = 0.93; main effect of group, *F* () = 0.066, *p* = 0.80) neurons didn’t affect freezing during extinction and retrieval. **(E)** Schematic of retroAAV-Cre injection into the vmPFC, combined with AAV injection encoding Cre-dependent ChrimsonR or control fluorophore into the RE or MD. **(F)** Representative images showing ChrimsonR-tdTomato expression. White bar = 200 μm. **(G**, **H)** 10 Hz stimulation of vmPFC-projecting **(G)** RE (Extinction, interaction, *F*(5, 115) = 0.63, *p* = 0.68; main effect of group, *F* (1, 23) = 0.10, *p* = 0.75. Extinction retrieval, interaction, *F* (2, 48) = 0.95, *p* = 0.39; main effect of group, *F* (1, 23) = 0.99, *p* = 0.33) or **(H)** MD (Extinction, interaction, *F*(5,80) = 0.50, *p* = 0.77; main effect of group, *F* (1, 16) = 0.0085, *p* = 0.93. Extinction retrieval, interaction, *F* (2, 32) = 1.79, *p* = 0.18, main effect of group: *F* (1, 16) = 0.31, *p* = 0.58) neurons had no effect on freezing during extinction and retrieval. All error bars indicate SEM across subjects. Numbers in brackets represent sample size. Two-way repeated measures ANOVA

Based on the findings that vmPFC-projecting RE neurons respond to CS+ during extinction, we asked whether this neuronal population plays a critical role in freezing behavior during extinction learning. To this end, we injected retroAAV-Cre into the vmPFC followed by the injection of Cre-inducible Jaws-GFP or control into either RE or MD (Fig. 4A and B). Mice underwent aversive conditioning followed by extinction, during which laser illumination was applied through implanted optic fibers during the CS period.

For vmPFC-projecting RE neurons, no significant effect on freezing was observed during extinction (Fig. 4C, two-way repeated measures ANOVA, *trial x group*, *F*(5, 95) = 1.29, *p* = 0.28; main effect of group, *F* (1, 19) = 0.024, *p* = 0.88). Similarly, during extinction retrieval, both groups displayed an equivalent level of freezing (*trial x group*, *F* (2, 38) = 2.25, *p* = 0.12; main effect of group, *F* (1, 19) = 0.0023, *p* = 0.96). Likewise, optogenetic inhibition of vmPFC-projecting MD neurons caused no significant effects on freezing during extinction (Fig. 4D, two-way repeated measures ANOVA, *trial x group*, *F*(5, 70) = 0.84, *p* = 0.52; main effect of group, *F* (1, 14) = 0.47, *p* = 0.50), as well as extinction retrieval (two-way repeated measures ANOVA, *trial x group*, *F* (2, 28) = 0.072, *p* = 0.93; main effect of group, *F* (1, 14) = 0.066, *p* = 0.80).

Given that both vmPFC-projecting RE and MD neurons respond to aversive events, we next aimed to examine whether stimulation of these circuits could disrupt extinction learning. To test this hypothesis, mice were injected with retroAAV-Cre into the vmPFC and an AAV encoding either a Cre-dependent excitatory, red light-activated opsin (AAV-hSyn-flex-ChrimsonR-tdTomato) or a control fluorescent protein (AAV-hSyn-flex-tdTomato) into the RE or MD, along with implantation of the optic fibers (Fig. 4E and F). In vmPFC-projecting RE neurons, optogenetic activation during the CS period failed to affect freezing behavior (Fig. 4G, two-way repeated measures ANOVA, *trial x group*, *F*(5, 115) = 0.63, *p* = 0.68; main effect of group, *F* [1, 23] = 0.10, *p* = 0.75). Similarly, no significant differences were observed during extinction retrieval (*trial x group*, (*F* [2, 46] = 0.95, *p* = 0.39; main effect of group, *F* [1, 23] = 0.99, *p* = 0.33). For vmPFC-projecting MD neurons, two-way repeated measures ANOVA revealed no significant differences on freezing during extinction (Fig. 4H, *trial x group*, *F*(5,80) = 0.50, *p* = 0.77; main effect of group: *F* [1, 16] = 0.0085, *p* = 0.93), as well as extinction retrieval (*trial x group*, *F* [2, 32] = 1.79, *p* = 0.18; main effect of group: *F* [1, 16] = 0.31, *p* = 0.58). Collectively, neither optogenetic activation nor inactivation of vmPFC-projecting neurons in the RE or MD affect freezing behavior during extinction and retrieval under the current experimental conditions.

## Discussion

Our findings reveal distinct anatomical properties and neuronal representations of limbic thalamic neurons projecting to the vmPFC, highlighting their roles in aversive memory formation and extinction. We demonstrated that neurons projecting to the vmPFC or dmPFC are topologically segregated within the RE and MD. Fiber photometry revealed that both vmPFC-projecting RE and MD neurons respond to a foot shock, whereas vmPFC-projecting RE, but not vmPFC-projecting MD, neurons develop a response to shock-associated cue during aversive conditioning. During extinction learning, vmPFC-projecting RE neurons displayed a biphasic response to the shock-associated cue, characterized by an initial increase in activity followed by suppression. Notably, the initial increased response was prominent during the early, but not late, phase of extinction. These findings suggest that RE neurons send aversive cue information to the vmPFC.

In line with the previous study [35, 36], we found that dmPFC-projecting neurons in mice are predominantly localized in the lateral RE and MD, whereas vmPFC-projecting neurons are distributed in the medial RE and MD. RE neurons send projections to the limbic and cortical areas [37, 38]. Among these, we identified a small fraction of RE neurons projecting to both vmPFC and dmPFC. In rats, Verela et al. reported that a small subset of RE neurons send collaterals to both mPFC and ventral hippocampus [20], suggesting that a distinct RE population primarily projects to the vmPFC. As RE neurons also send projections to basolateral and medial amygdala [31, 37], vmPFC-projecting RE neurons could send collaterals to these areas. In contrast to the RE, MD neurons innervate mostly cortical areas with some innervations to the basolateral amygdala [38–41]. Similar to the RE, we discovered that distinct RE populations primarily project to the vmPFC or dmPFC, though both of them could have other axon collaterals.

To our knowledge, this is the first study on rodents that show the increased activity of vmPFC-projecting RE neurons in response to an aversive foot shock and cue. Consistent with this notion, the human study has demonstrated that the RE showed elevated activation when young participants were exposed to negative stimuli [42]. On the other hand, it has been reported that some RE neurons exhibit CS-evoked responses to an extinguished CS during extinction retrieval relative to renewal [42]. In addition, RE bulk CA_2+_ signals increase at the freezing cessation during extinction of aversive contextual memory [43]. These studies indicate that subsets of RE neurons respond to extinguished CS or behavior transition from defensive state. Further studies are needed to understand whether the majority of vmPFC-projecting RE neurons respond to aversive events.

The MD is a component of the medial pain system, which is considered to represent the affective or emotional dimension of pain [44]. Activation of MD inputs onto the anterior cingulate cortex, a part of dmPFC, has been shown to elicit conditioned place aversion in neuropathic pain models [45]. Beyond its role in the medial pain system, we discovered that vmPFC-projecting MD neurons, distinct from dmPFC-projecting neurons, also respond to an aversive foot shock. This finding highlights the role of MD neurons in conveying painful information to not only the mPFC but also to the vmPFC. In addition, the MD modulates extinction through its firing modes, with tonic firing facilitating aversive memory extinction but burst firing suppressing it [27]. Although the observed CA_2+_ activity in vmPFC-projecting MD neurons after CS onset at the early phase of extinction was not statistically significant, it could reflect burst firing activity, consistent with its potential role in inhibiting extinction learning.

As different subpopulations in the vmPFC exert opposite effects in modulating fear and anxiety [46], the vmPFC could have different neural populations which promote and suppress aversive memory extinction. Indeed, electrophysiological studies have identified neurons in the vmPFC that respond to the CS during either early or late phase of extinction [11, 47]. Furthermore, it’s been shown that the vmPFC is capable of controlling both promotion and suppression of reward seeking via different neural ensembles [48]. Thus, observed cue-onset increase in activity of vmPFC-RE or MD neurons at the early phase of extinction could enhance CS+ E responsive neurons in the vmPFC which suppress extinction.

Though our results showed no evidence of significant effects on freezing behavior by optogenetic manipulations, CS evoked vmPFC-projecting RE neuronal response could be involved in other functions such as consolidation of aversive memory [49], reconsolidation [50], and trace fear [51]. Additionally, several studies have highlighted the importance of temporal coding in the RE and MD. It has been shown that frequency-specific optogenetic or electrical stimulation of these nuclei can significantly influence behavioral and neural outcomes [52]. These findings suggest that future experiments employing frequency-specific manipulations may provide deeper insights into the functional roles of RE and MD in extinction.

Overall, our findings reveal distinct anatomical and functional properties of vmPFC-projecting neurons in the RE and MD, with vmPFC-projecting RE neurons uniquely encoding aversive cues during both conditioning and extinction. This study expands our understanding of the RE’s and MD’s roles in aversive memory processes. Future studies integrating the advanced techniques such as frequency manipulation and single cell recording will be needed for understanding the functional roles of these thalamic nuclei in conveying aversive signals to the vmPFC.

## Electronic supplementary material

Below is the link to the electronic supplementary material.

Supplementary Material 1

## Data Availability

The data that support the finding of this study are available upon reasonable request.

## Abbreviations

vmPFC: Ventromedial prefrontal cortex
dmPFC: Dorsomedial prefrontal cortex
RE: Reuniens thalamic nucleus
MD: Mediodorsal thalamic nucleus
CTB: Cholera toxin subunit B
CS: Conditioned stimulus
US: Unconditioned stimulus
ITI: Intertrial interval
CS+ E: CS+ at the early extinction trials
CS+ L: CS+ at the late extinction trials
