# Supplementary material for: Distinct neural responses of ventromedial prefrontal cortex-projecting nucleus reuniens neurons during aversive memory extinction
Source: Mol Brain. 2025 Mar 5;18:18. doi: 10.1186/s13041-025-01185-y (PMC11881366; doi:10.1186/s13041-025-01185-y)
Supplement: Supplementary file 1 — Supplementary Material 1 [file 13041_2025_1185_MOESM1_ESM.docx]

**Supplementary Figures**


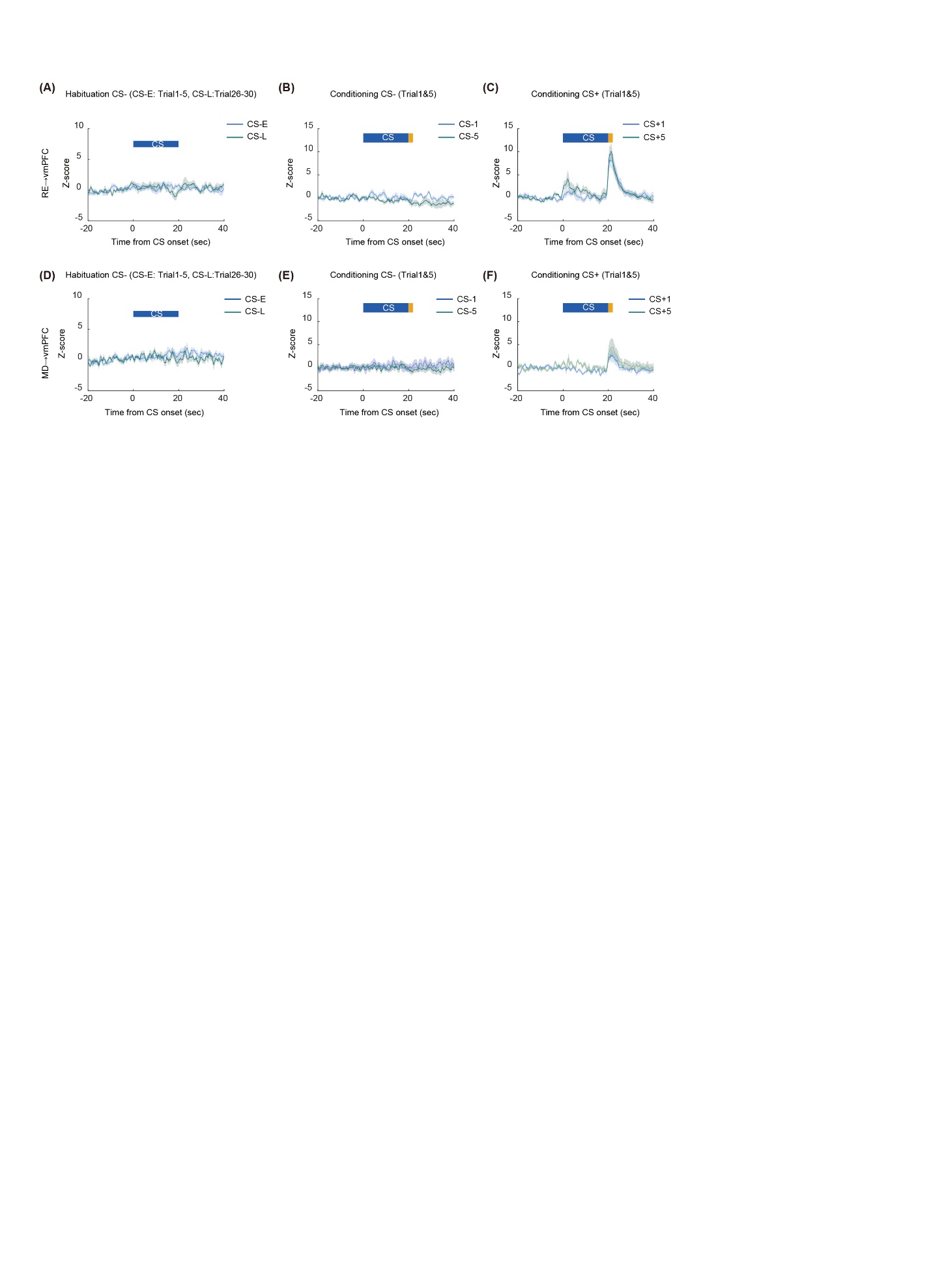


**Supplementary Figure 1. Mean Z-scored calcium activity of vmPFC-projecting RE or MD neurons during habituation and aversive conditioning.**

**(A, D)** Mean Z-scored calcium activity of vmPFC-projecting (A) RE or (D) MD neurons from a population of mice in response to CS- during early and late trials of habituation. **(B, E)** Mean Z-scored calcium activity of vmPFC-projecting (B) RE or (E) MD neurons from a population of mice in response to CS- at trial 1 and 5 during aversive conditioning. **(C, F)** Mean Z-scored calcium activity of vmPFC-projecting (B) RE or (E) MD neurons from a population of mice in response to CS+ at trial 1 and 5 during aversive conditioning. Shaded region denotes standard mean errors (n = 7). At the top of each plot, blue and orange lines denote CS and US period, respectively.


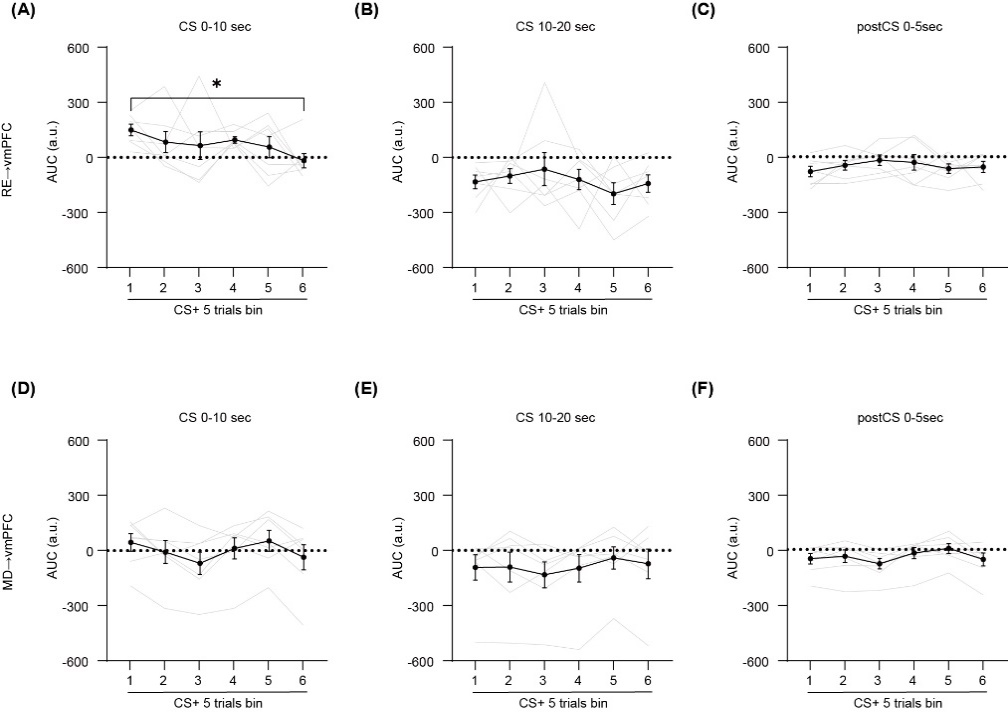


**Supplementary Figure 2. Mean AUC of vmPFC-projecting RE or MD neurons during extinction.**

**(A, D)** Mean Z-score AUC at every 5 trials bin during the 10sec after CS onset in (A) RE and (D) MD. **(B, E)** Mean Z-score of the AUC at every 5 trials bin during the 10sec before CS offset in (B) RE and (E) MD. (C, F) Mean Z-score of the AUC for each trial during the 5sec after CS offset in the (C) RE and (F) MD. All error bars indicate SEM across subjects (n = 7). One-way repeated measures ANOVA followed by Dunnett’s test.
